# MERTK Is a Potential Therapeutic Target in Ewing Sarcoma

**DOI:** 10.3390/cancers16162831

**Published:** 2024-08-12

**Authors:** Sherri K. Smart, Tsz Y. Yeung, M. Olivia Santos, Leon F. McSwain, Xiaodong Wang, Stephen V. Frye, H. Shelton Earp, Deborah DeRyckere, Douglas K. Graham

**Affiliations:** 1 Aflac Cancer and Blood Disorders Center, Children’s Healthcare of Atlanta, Atlanta, GA 30322, USA; sherri.kay.smart@emory.edu (S.K.S.); jessica.yeung@emory.edu (T.Y.Y.); leon.foy.mcswain@emory.edu (L.F.M.); deborah.deryckere@emory.edu (D.D.); 2 Department of Pediatrics, Emory University School of Medicine, Atlanta, GA 30322, USA; 3 Duke University School of Medicine, Durham, NC 27710, USA; maria.santos@duke.edu; 4 Center for Integrative Chemical Biology and Medicinal Chemistry, UNC Eshelman School of Pharmacy, Chapel Hill, NC 27599, USA; xiaodonw@email.unc.edu (X.W.); svfrye@email.unc.edu (S.V.F.); 5 UNC Lineberger Comprehensive Cancer Center, University of North Carolina at Chapel Hill, Chapel Hill, NC 27599, USA; shelton_earp@med.unc.edu; 6 Departments of Medicine and Pharmacology, School of Medicine, University of North Carolina at Chapel Hill, Chapel Hill, NC 27599, USA

**Keywords:** ewing sarcoma, MERTK, MRX-2843, BCL-2, BCL-XL, small-molecule inhibitor

## Abstract

**Simple Summary:**

Ewing sarcoma family tumors are the second most common bone cancer affecting adolescents and young adults. Outcomes are poor in patients with metastatic or relapsed disease, and new treatments are urgently needed. MERTK is a protein found in leukemia, melanoma, lung cancer, and other cancer types where it promotes cancer cell survival and resistance to therapies. MRX-2843 is a new drug that targets MERTK and is currently in clinical trials. We show that MERTK is present on Ewing sarcoma cells and that MRX-2843 has therapeutic activity against these cells. MRX-2843 is even more effective against Ewing sarcoma cells when combined with other drugs, venetoclax or navitoclax, that target a protein called BCL-2. These results suggest that MRX-2843 could be useful to treat Ewing sarcoma in patients.

**Abstract:**

Outcomes are poor in patients with advanced or relapsed Ewing sarcoma (EWS) and current treatments have significant short- and long-term side effects. New, less toxic and more effective treatments are urgently needed. MER proto-oncogene tyrosine kinase (MERTK) promotes tumor cell survival, metastasis, and resistance to cytotoxic and targeted therapies in a variety of cancers. *MERTK* was ubiquitously expressed in five EWS cell lines and five patient samples. Moreover, data from CRISPR-based library screens indicated that EWS cell lines are particularly dependent on MERTK. Treatment with MRX-2843, a first-in-class, MERTK-selective tyrosine kinase inhibitor currently in clinical trials, decreased the phosphorylation of MERTK and downstream signaling in a dose-dependent manner in A673 and TC106 cells and provided potent anti-tumor activity against all five EWS cell lines, with IC_50_ values ranging from 178 to 297 nM. Inhibition of MERTK correlated with anti-tumor activity, suggesting MERTK inhibition as a therapeutic mechanism of MRX-2843. Combined treatment with MRX-2843 and BCL-2 inhibitors venetoclax or navitoclax provided enhanced therapeutic activity compared to single agents. These data highlight MERTK as a promising therapeutic target in EWS and provide rationale for the development of MRX-2843 for the treatment of EWS, especially in combination with BCL-2 inhibitors.

## 1. Introduction

The Ewing sarcoma family of tumors comprises Ewing sarcoma (EWS) and primitive neuroectodermal tumors (PNETs), and is characterized by chimeric proteins derived from the *EWSR1* gene [1,2]. EWS is the second most common bone sarcoma and affects approximately 200 adolescents and young adults each year in the United States [1]. There is a slight male predominance, and EWS most commonly affects white non-Hispanic and Hispanic populations. Approximately 20% of tumors arise in an extraosseous location [1]. The hallmark of EWS is the EWS-FLI1 fusion protein, which acts as a transcription factor, driving aberrant gene expression. Approximately 85–90% of EWS cases have an EWS-FLI1 fusion, and ~10% have an EWS-ERG fusion [1]. Other rare EWS fusion proteins have been reported [3]. EWS is an aggressive tumor and is currently treated with a combination of chemotherapy, surgery and/or radiation therapy. Multiple toxicities are associated with these therapies, including, but not limited to, acute side effects (severe neutropenia, opportunistic infections, mucositis) and possible long-term complications including cardiac toxicity, ototoxicity, renal dysfunction, infertility, neurocognitive dysfunction, and secondary malignancy [1,2,4]. The five-year overall survival rates for patients with localized disease are over 80%, but less than 40% for patients with metastatic disease at diagnosis and even lower at <20% for those with relapsed or refractory disease [1,5]. Minimal therapeutic advances have occurred in the last few decades, and new, more targeted and less toxic therapies are urgently needed. Receptor tyrosine kinase inhibitors (TKIs) have shown promise in EWS and other bone sarcomas [6,7].

One promising target is MER tyrosine kinase (MERTK). MERTK is a member of the TAM family (TYRO3, AXL, MERTK) of receptor tyrosine kinases and is aberrantly or ectopically expressed in a spectrum of human cancers, where it functions to promote tumor cell survival, metastasis and chemoresistance [8]. The best characterized ligands are GAS6, PROTEIN S (PROS1) and GALECTIN 3 (LGALS3). MERTK and TYRO3 are stimulated by all three ligands, but AXL is only stimulated by GAS6 [9]. Ligand binding to TAM family receptors activates downstream signaling pathways critical for cancer, including the JAK-STAT, MAPK-ERK, and PI3K-AKT-mTOR pathways and pro-survival pathways involving BCL-2 and SURVIVIN [10,11]. MERTK is also expressed on macrophages and other innate immune cells where it can function to suppress anti-tumor immunity [9]. Thus, agents targeting MERTK are expected to provide therapeutic activity via multiple mechanisms, including direct tumor cell killing, inhibition of metastasis, and activation of anti-tumor immunity. Moreover, MERTK inhibition may be particularly effective in combination with chemotherapy and targeted agents [8,12,13,14]. 

MRX-2843 is a first-in-class, MERTK-selective and orally bioavailable receptor tyrosine kinase inhibitor developed in our laboratory in collaboration with other scientists [15,16,17]. MRX-2843 and related MERTK TKIs mediate direct tumor cell killing in preclinical models and promote sensitivity to chemotherapy in vitro and in murine models of leukemia, melanoma, lung cancer, and many other malignancies [12,15,17,18,19,20,21,22]. MRX-2843 is also being advanced in multiple phase I trials, including studies in (1) adults with relapsed/refractory advanced and/or metastatic solid tumors (NCT03510104), (2) adolescents and adults with relapsed/refractory acute myeloid leukemia (AML), acute lymphoid leukemia (ALL), or mixed-phenotype acute leukemia (NCT04872478), and (3) combined with Osimertinib for treatment of advanced EGFR mutant non-small cell lung cancer (NCT04762199).

Here, we explored the potential utility of MERTK as a therapeutic target and evaluated MRX-2843 as a therapeutic agent in EWS, both alone and in combination with B-cell lymphoma 2 (BCL-2) family inhibitors. Previous studies have shown that the chimeric transcription factor EWS-FLI1 induces BCL-2 expression, promoting cancer cell survival [23]. The BCL-2 family includes anti-apoptotic proteins (BCL-2, BCL-XL, MCL-1, BCL-W, BFL-1), pro-apoptotic effector proteins (BAX, BAK, BOK), and pro-apoptotic BH3-only proteins (BAD, BIM, BID, NOXA, PUMA, BIK, and HRK) [24,25]. Overexpression of pro-survival BCL-2 family proteins occurs in numerous cancer types and leads to cancer cell survival and resistance to therapies [24,25,26,27,28]. Of particular importance in pediatric solid tumors are the BCL-2 proteins BCL-XL and MCL-1 [26,27,29].

## 2. Materials and Methods

### 2.1. CERES Dependency

Publicly available CRISPR-CAS9 knock-out dependency data from the Broad Institute’s Cancer Dependency Map (DepMap) portal Avana 20Q4 database at https://depmap.org/portal/ (accessed on 19 January 2021) were queried for dependence on TAM family receptors and ligands. CERES dependency is a computational method of estimating gene dependency in genome-scale CRISPR-Cas9 essentiality screens that decreases false positive results from copy-number-specific effects due to Cas9-mediated DNA cleavage [30]. A lower CERES score indicates greater functional dependence, with a score of −1 indicating an effect comparable to the median value for all pan-essential genes and a score of 0 indicating no significant reduction in cell proliferation or viability [30].

### 2.2. RNA Expression Data

Data describing MERTK RNA expression in EWS cell lines were obtained from the DepMap Portal Avana 20Q4 at https://depmap.org/portal/ (accessed on 19 January 2021). The RNASeq files were aligned with Spliced Transcripts Alignment to a Reference (STAR) and quantified with RNA-Seq by Expectation–Maximation (RSEM), then normalized for transcripts per million (TPM). Data are reported as log2(TPM + 1). The data for MERTK RNA expression in EWS patient samples were derived from the pediatric cancer data portal (PeCan) at https://pecan.stjude.cloud/ (accessed on 8 June 2023). Fragments per Kilobase of transcript per Million mapped reads (FPKM) were log2(FPKM + 1) normalized. Raw data files are available under accession number dbGaP phs000424.v8.p2.

### 2.3. Cell Culture and Reagents

The SK-ES-1 and RD-ES EWS cell lines were purchased from ATCC. The A673 EWS cell line was obtained from Dr. Andrew L. Hong (Emory University/CHOA, Atlanta, GA, USA). TC106 EWS cells were obtained from T. Triche (Children’s Hospital Los Angeles, Los Angeles, CA, USA). The TC32 PNET cell line was obtained from M. Tsokos (NCI, Bethesda, MD, USA) or Carol Thiele (NCI, Bethesda, MD, USA). U2OS osteosarcoma cells were purchased from ATCC (Manassas, VA, USA). TC106, TC32, RD-ES cell lines were cultured in RPMI medium, A673 was cultured in DMEM, and U2OS and SK-ES-1 were cultured in McCoy medium. Media was supplemented with 10% or 15% (TC106, SK-ES-1) fetal bovine serum and 1% penicillin–streptomycin (complete growth medium). All cell lines were DNA sequence verified by short tandem repeat (STR) analysis and tested negative for mycoplasma. Trypsin-EDTA (Invitrogen, Thermo Fisher Scientific, Waltham, MA, USA) or mechanical dissociation with a cell scraper was used to collect adherent cell lines. Stock strains thawed from liquid nitrogen storage were maintained in cell culture for no longer than three months.

### 2.4. Small Molecule Inhibitors

MRX-2843 was synthesized as previously described [15]. Venetoclax was obtained from LC Laboratories (Woburn, MA, USA), and navitoclax was obtained from MedChem Express (Monmouth Junction, NJ, USA). Stock solutions were prepared in DMSO (Sigma-Aldrich, St. Louis, MO, USA).

### 2.5. Immunoblot Analysis

Cell lysates were prepared in 150 mmol/L NaCl, 50 nmol/L HEPES pH 7.5, 10 mmol/L EDTA, 10% glycerol, 1% Triton X-100, and a protease inhibitor cocktail (ref 5892970001, Millipore Sigma, Darmstadt, Germany). Proteins were resolved on Tris-Glycine SDS-PAGE gels (Invitrogen, Thermo Fisher Scientific, Waltham, MA, USA) and transferred to nitrocellulose membranes. Membranes were blocked with 5% milk in Tris-buffered saline with 0.1% tween 20 (TBST) and incubated with antibodies specific to proteins of interest. Proteins were visualized on X-ray film using Western Lightning Plus ECL reagent (Perkin Elmer, Waltman, MA, USA). Membranes were stripped and reprobed for total protein or loading control. The following antibodies were used: MERTK (ab52968, Abcam, Cambridge, UK), AXL (AF154, R&D Systems, Minneapolis, MN, USA), TYRO3 (5585S, Cell Signaling, Danvers, MA, USA), PROS1 (A0384, Dako, Agilent Technologies Inc., Santa Clara, CA, USA), LGALS3 (ab2785, Abcam, Cambridge, UK), ACTIN (sc-47778, Santa Cruz Biotechnology, Dallas, TX, USA), and the remainder from Cell Signaling (Danvers, MA, USA): phosphorylated ERK1/2 (9106), ERK1/2 (9102), phosphorylated STAT6 (93625S), STAT6 (93625) and SURVIVIN (2808). The secondary antibodies used for immunoblotting were goat anti-rabbit IgG-HRP (170-6515, Bio-Rad Technologies, Hercules, CA, USA), goat anti-mouse IgG-HRP (170-6516, Bio-Rad Technologies, Hercules, CA, USA), and mouse anti-goat IgG-HRP (sc-2354, Santa Cruz Biotechnology, Dallas, TX, USA). The relative protein levels were quantified by densitometry using ImageJ software, version 1.54j (NIH, Bethesda, MD, USA). Blots are representative of at least three independent experiments.

### 2.6. Immunoblot Analysis of MERTK

Approximately 1 million cells were cultured in a 6-well plate and allowed to adhere overnight, then treated with MRX-2843 or an equivalent volume of DMSO vehicle for 1 h. Cells were treated with pervanadate phosphatase inhibitor, and MERTK was immunoprecipitated as previously described [15]. MERTK antibody (MAB8912, R&D Systems, Minneapolis, MN, USA) was used for immunoprecipitation and phosphorylated MERTK (Y749, Y753, Y754; PhosphoSolutions, Aurora, CO, USA) and MERTK (ab52968, Abcam, Cambridge, UK) antibodies were used for immunoblotting.

### 2.7. Downstream Signaling Assays

For assessment of ERK and STAT6 signaling, cells (TC106 = 500 K per 6 well in 1.5 mL medium; A673 200 K per 6 well in 1.5 mL) were cultured in complete growth medium overnight and in serum-free medium for an additional 2 h. MRX-2843 or vehicle was added during the last 90 min of culture and GAS6 or vehicle was added during the last 12 min. For assessment of survivin, cells were cultured in complete growth medium with MRX-2843 or vehicle for 72 h.

### 2.8. Cell Density Assay

Cells were cultured in a 96-well plate at 30,000 (A673) or 35,000 (TC106) cells/well in complete growth media overnight, then treated with MRX-2843, venetoclax (VEN) or navitoclax (NAV), a combination of MRX-2843 and VEN or NAV, or DMSO vehicle for 72 h. The relative cell densities were determined with CellTiter-Glo reagent (Promega, Madison, WI, USA). Luminescence was measured using a plate reader. Data are derived from at least three independent experiments.

### 2.9. Clonogenic Assay

A673 cells were cultured overnight in complete growth medium at a density of 300 cells/well in a 24-well plate and then treated with the indicated concentrations of MRX-2843 or an equivalent volume of DMSO control for 6–8 days. Colonies were stained with 0.2% crystal violet in 25% methanol for 4 min, then dried for 1–2 days and counted using a GelCount colony counter (Oxford Optronix, Oxfordshire, UK).

### 2.10. Statistics

The Wilcoxon rank-sum test was utilized for CERES dependency score comparisons between EWS cells and all other cell types, given the nonparametric dataset. Data from the cell density assays were evaluated using one- or two-way ANOVA with Bonferroni’s or Tukey’s post-tests, respectively, to determine statistical differences (*p* < 0.05). Statistical analysis was performed using Prism 9 software, version 9.5.1 (GraphPad Software, San Diego, CA, USA).

## 3. Results

### 3.1. Ewing Sarcoma Cell Lines Are Functionally Dependent on MERTK

**Figure 1:**
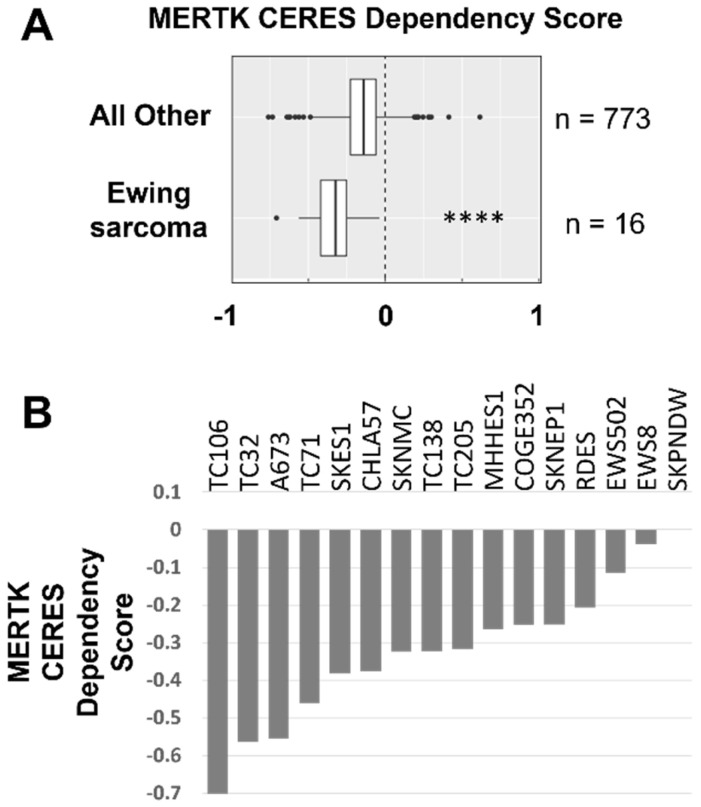
Ewing sarcoma cell lines are functionally dependent on MERTK. (**A**) MERTK CERES gene dependency scores were determined using CRISPR-CAS9 knockout data obtained from the Broad Institute’s Cancer Dependency Map (DepMap) portal Avana 20Q4 database. A lower CERES score indicates greater functional dependence, with a score of −1 indicating an essential gene and a score of 0 indicating no significant reduction in cell density. Data are depicted as box plots, with median CERES dependency score flanked by quartile 1 and 3 and whiskers on either side of box showing distribution of data. Outliers are indicated with dots. MERTK CERES Dependency scores demonstrate enhanced dependency on MERTK in EWS cell lines relative to all other cell lines tested (**** *p* = 0.00001, Wilcoxon rank-sum test). (**B**) CERES dependency scores in individual EWS cell lines.

To evaluate the potential roles of MERTK in EWS, we analyzed publicly available data from genome-wide CRISPR-Cas9-based knockout library screens from the Broad Institute Cancer Dependency Map (DepMap) portal. The DepMap portal includes a multitude of cell lines derived from solid tumors, brain tumors, and hematologic malignancies. Changes in cell density in response to target knockout were determined, with a more negative score indicating greater functional significance. Specifically, a score of −1 indicates an essential gene and a score of 0 indicates no significant reduction in the relative cell density in response to target depletion [30]. EWS cell lines (*n* = 16) were particularly dependent on MERTK, with a lower CERES score in EWS relative to all other cancer cell lines (*n* = 773) (*p* = 1 × 10^−5^) (Figure 1 and Figure S1). The cell lines with the highest level of dependency on MERTK (CERES score less than −0.5) were TC106, TC32, and A673. There was also a trend toward decreased CERES scores for the TAM-family kinase TYRO3 in EWS compared to all other cancer cell lines, but the difference was not statistically significant (*p* = 0.07) (Supplemental Figure S2). The TAM-family kinase AXL and ligands GAS6, PROS1 and LGALS3 did not demonstrate functional significance (Supplemental Figure S2).

### 3.2. MERTK Is Expressed in Ewing Sarcoma

**Figure 2:**
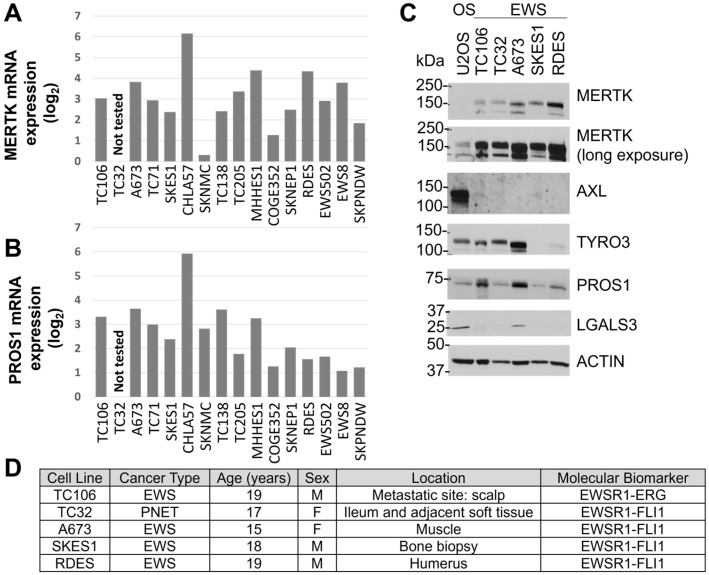
MERTK is expressed in Ewing sarcoma (EWS) cells. (**A**) Relative MERTK and (**B**) PROS1 mRNA levels in EWS cell lines were determined with RNA sequencing data obtained from the Broad Institute’s Cancer Dependency Map (DepMap) portal Avana 20Q4 database. (**C**) Relative protein expression of TAM family members (TYRO3, AXL, MERTK) and ligands (PROS1, LGALS3) was determined via immunoblotting of lysates from EWS cell lines. The osteosarcoma (OS) cell line U2OS was used as a positive control. ACTIN was used as a loading control. Images are representative of at least three independent experiments. (**D**) Characteristics of EWS cell lines are depicted in panel (**C**).

Publicly available data from the DepMap Portal were queried for MERTK expression in Ewing family cell lines. MERTK mRNAs were detected at varying levels in 15 of 16 EWS cell lines (Figure 2A). MERTK ligand PROS1 mRNA expression was also detected in 15 of 16 EWS cell lines (Figure 2B). Relative TAM family protein (TYRO3, AXL, MERTK) and ligand (LGAL3, PROS1) levels were determined by immunoblotting in a panel of five EWS/PNET family cell lines. Consistent with the gene expression data, all five EWS cell lines tested (TC106, TC32, A673, SK-ES-1, and RD-ES) expressed relatively high levels of MERTK protein compared to the weaker expression of MERTK in the osteosarcoma cell line U2OS, which was used as a positive control for TAM kinase expression (Figure 2C). The MERTK ligand PROS1 was also expressed in all cell lines tested, suggesting the possibility of an autocrine or paracrine feedback loop resulting in MERTK activation in EWS cells (Figure 2C). While robust levels of AXL were detected in U2OS, AXL was present at very low levels or absent in EWS cell lines. TYRO3 and LGALS3 were differentially expressed across the panel of cell lines tested. Four of five cell lines expressed the EWS-FLI1 fusion protein, while TC106 expressed the EWS-ERG fusion protein (Figure 2D).

**Figure 3:**
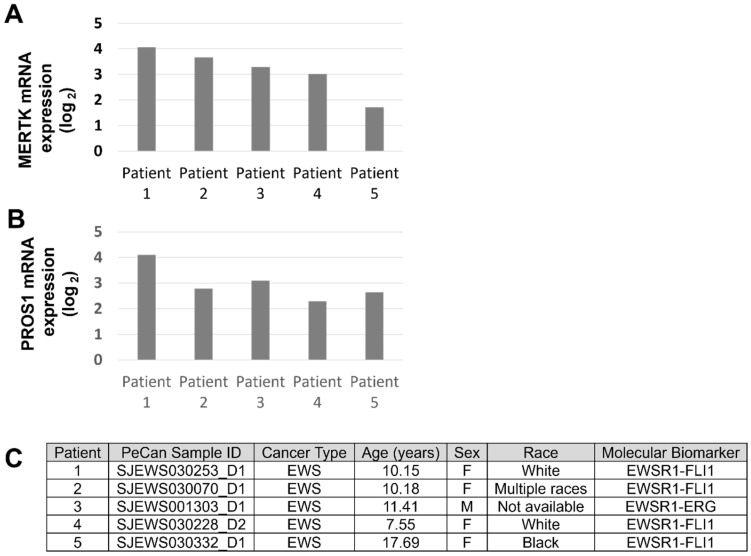
MERTK is expressed in Ewing sarcoma patient samples. RNA sequencing data were obtained from the pediatric cancer (PeCan) database and relative levels of (**A**) MERTK and (**B**) PROS1 mRNA in EWS patient samples were determined. (**C**) Characteristics of EWS patient samples depicted in panel (**A**,**B**).

The PeCan database was also queried to evaluate *MERTK* mRNA expression in pediatric patient samples. Although samples from only five patients were available, *MERTK* mRNA was detected in all five (Figure 3A). PROS1 mRNA was also expressed in all five patient samples (Figure 3B). Four of five tumors expressed an EWS-FLI1 fusion protein, and the remaining tumor expressed an EWS-ERG fusion protein (Figure 3C).

### 3.3. MRX-2843 Inhibits MERTK Kinase Activity and Has Potent Anti-Tumor Activity in EWS Cells

**Figure 4:**
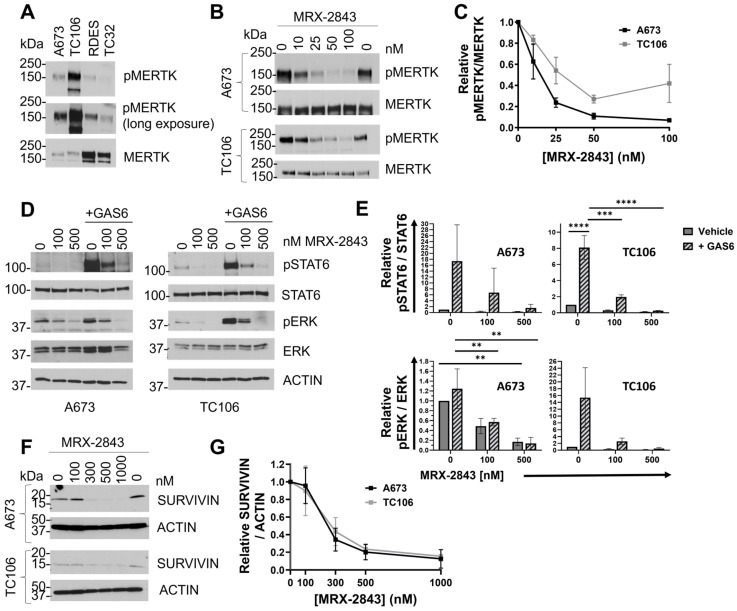
MRX-2843 mediates dose-dependent inhibition of MERTK kinase and downstream signaling. (**A**,**B**) Lysates were prepared from cultures treated with pervanadate phosphatase inhibitor for 3 min and, where indicated, with MRX-2843 or vehicle for 1 h. MERTK was immunoprecipitated and phosphorylated (denoted by *p*) and the total proteins were detected by immunoblot. Long and short exposures are shown. (**B**) A673 and TC106 cells were treated with MRX-2843. (**C**) Relative pMERTK/MERTK levels were quantified by densitometry. Mean values and standard error derived from three to five independent experiments are shown. IC_50_ values and 95% confidence intervals (C.I.) were calculated by nonlinear regression. (**D**,**E**) Serum-starved cultures were treated with MRX-2843 or vehicle for 90 min and with GAS6 conditioned medium for the last 12 min as indicated. Cell lysates were prepared and phosphorylated/activated (denoted by *p*) and total ERK1/2 and STAT6 levels were detected by immunoblot. Mean values and standard errors from three independent experiments are shown (** *p* < 0.01, *** *p* < 0.001, **** *p* < 0.0001, two-way ANOVA). (**E**) Relative pERK/ERK and pSTAT6/STAT6 levels were determined by densitometry. (**F**) Lysates were prepared from cultures treated with MRX-2843 or vehicle for 72 h, and survivin protein levels were determined by immunoblot. Actin was used as a loading control. (**G**) Relative levels of SURVIVIN/ACTIN were quantified by densitometry. Mean values and standard errors from three independent experiments are shown.

**Figure 5:**
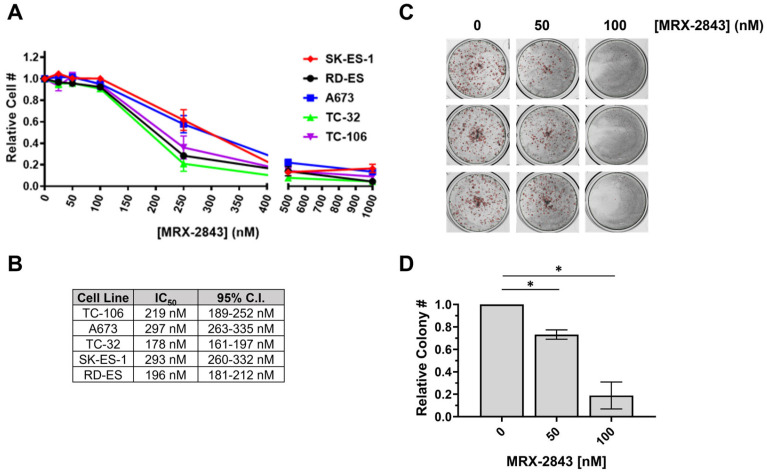
MRX-2843 has potent anti-tumor activity against EWS cell lines. (**A**,**B**) EWS cells were cultured with MRX-2843 or vehicle for 72 h and relative viable cell numbers were determined using CellTiter-Glo reagent. (**A**) Mean values and standard errors from 3–4 independent experiments. (**B**) IC_50_ values and 95% confidence intervals (C.I.) were calculated by non-linear regression from the data shown in panel (**A**). (**C**,**D**) A673 cells were cultured at low density with MRX-2843 or vehicle for 6–8 days, and colonies were counted. (**C**) Representative cultures are depicted. (**D**) Mean relative colony numbers and standard errors from three independent experiments are shown (* *p* < 0.05, unpaired *t* test).

Active, phosphorylated MERTK (pMERTK) was consistently detected in a panel of EWS/PNET cell lines, although relative total and phosphoprotein levels varied between experiments (Figure 4A and Supplemental Information, immunoblots for Figure 2C and Figure 4A). The reasons for these fluctuations are unknown, but changes in MERTK levels in response to glucose deprivation have been previously reported [31]. A673 and TC106 cell lines were selected for further characterization because they had the lowest CERES dependency scores and the highest relative phosphorylated to total MERTK ratios among the cell lines tested. Treatment with MRX-2843 decreased phosphorylation of MERTK in a dose-dependent manner in A673 and TC106 cells (Figure 4B), with IC_50_ values of 13.3 nM (95% C.I.; 8.3–17.9 nM) and 34.5 nM (95% C.I.; 13.3–119.7 nM), respectively (Figure 4C). Downstream signaling was also inhibited in cells treated with MRX-2843. ERK1/2 and STAT6 phospho-protein levels were reduced in A673 and TC106 cell lines after MRX-2843 treatment, with inhibition evident at concentrations <100 nM (Figure 4D,E). Survivin, an IAP family apoptosis inhibitor, was also decreased in cells treated with MRX-2843 compared to vehicle-treated cells (Figure 4F,G). Similarly, treatment with MRX-2843 resulted in a dose-dependent reduction in cell density relative to the vehicle in all five EWS cell lines (Figure 5A), with IC_50_ values ranging from 178 to 297 nM (Figure 5B). A673 cells were even more sensitive to MRX-2843 in clonogenic assays, where treatment with 100 nM MRX-2843 resulted in an 80% reduction in colony formation (Figure 5C,D).

### 3.4. MRX-2843 Provides Enhanced Therapeutic Efficacy in Combination with BCL-2 Inhibitors

**Figure 6:**
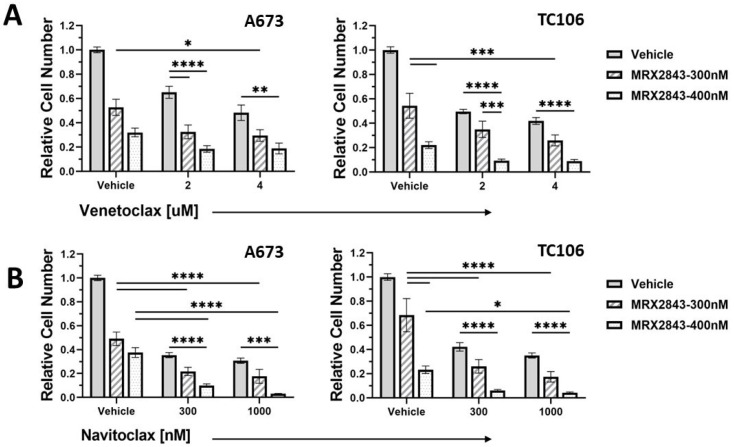
Combined treatment with MRX-2843 and BCL-2 inhibitors provides enhanced therapeutic efficacy compared to single agents in Ewing sarcoma cell cultures. A673 or TC106 cells were treated for 48 h with MRX-2843, a BCL-2 inhibitor (venetoclax (VEN, (**A**)) or navitoclax (NAV, (**B**))), MRX-2843 combined with VEN or NAV, or vehicle, and then the cells were stained with CellTiter-Glo reagent and the luminescence was measured. Cell numbers were calculated relative to the vehicle control. Means ± SEM were derived from 3–6 independent experiments (* *p* < 0.05, ** *p* < 0.01, *** *p* < 0.001, **** *p* < 0.0001, 2-way ANOVA). All treatments provided a significant decrease in tumor cell density relative to vehicle (*p* ≤ 0.0001).

BCL-2 is often upregulated in EWS cells, and BCL-2 inhibitors have been combined with agents representing several classes of drugs to enhance therapeutic efficacy against EWS cells [23,32,33,34]. Here, A673 and TC106 cells were treated with MRX-2843 and the BCL-2 inhibitors venetoclax or navitoclax alone and in combination, and relative cell densities were assessed to determine whether MRX-2843 and BCL-2 inhibitors can be similarly combined. Treatment with MRX-2843, venetoclax or navitoclax monotherapies significantly reduced tumor cell density compared to the vehicle in both cell lines (Figure 6, *p* < 0.0001). Additionally, combined treatment with MRX-2843 and the BCL-2 inhibitor venetoclax provided significantly enhanced therapeutic efficacy compared to MRX-2843 and/or venetoclax in both cell lines (Figure 6A). For instance, in A673 cultures treatment with 300 nM of MRX-2843 or 2 µM of venetoclax mediated respective reductions of 47.3% ± 6.7% and 34.9% ± 4.9% in cell density compared to the vehicle, while combined treatment with MRX-2843 and 2 µM of venetoclax reduced cell density by 67.5% ± 5.6% (Figure 6A, left panel). Higher concentrations of 400 nM MRX-2843 or 4 µM ventoclax reduced cell densities by 68.0% ± 3.6% and 51.8% ± 6.4%, respectively, while the combination provided an 81.6% ± 2.7% reduction in tumor cells. Similar results were observed in TC106 cultures, where the combination of 400 nM MRX-2843 with 4 µM venetoclax provided a 91.1% ± 1.4% reduction in the tumor cell number (Figure 6A, right panel). The BCL-2 and BCL-XL inhibitor navitoclax also enhanced therapeutic efficacy in combination with MRX-2843 (Figure 6B). In A673 cultures, treatment with 300 nM of MRX-2843 or 300 nM of navitoclax mediated 50.9% ± 5.7% and 64.6% ± 2.2% reductions in the cell density compared to the vehicle, while combined treatment reduced the cell density by 78.2% ± 3.5% (Figure 6B, left panel). Similarly, in TC106 cultures, treatment with 400 nM MRX-2843 or 1 µM navitoclax mediated 76.7% ± 3.1% and 65.0% ± 2.1% reductions in cell density, respectively, while combined treatment reduced the cell density by 95.8% ± 0.6% (Figure 6B, right panel). To evaluate the interactions between MRX-2843 and BCL-2 inhibitors, the expected additive effect was calculated for each combination based on the observed effects of the single agents, and these values were compared with the observed effects of the combination therapies. This analysis revealed an additive interaction between MRX-2843 and both BCL-2 inhibitors under most conditions tested (Supplemental Figure S3). The interaction between MRX-2843 and navitoclax in A673 cells was synergistic when higher concentrations of both drugs (400 nM MRX-2843 and 1000 nM navitoxlax) were combined (Supplemental Figure S3).

## 4. Discussion

Treatment of adolescents and young adults with metastatic, relapsed or refractory EWS remains a challenge, and the therapies that are currently used have high potential for significant long-term toxicity [1,2]. New strategies for treatment are needed, and molecularly targeted approaches are expected to have fewer side effects [6,7]. In this study, we demonstrated MERTK expression in EWS cell lines and patient samples. All of the cell lines and patient samples evaluated also expressed one or more MERTK ligands, suggesting autocrine activation in EWS cells to exploit MERTK cell survival and proliferation pathways. Biochemical studies demonstrated phosphorylation of MERTK in EWS cells, confirming that the kinase is active. Moreover, publicly available data from CRISPR-based library screens indicate that EWS cell lines are particularly dependent on MERTK. These data suggest that therapeutic strategies targeting MERTK may be particularly effective for the treatment of EWS.

MRX-2843 is a first-in-class MERTK-selective inhibitor that is currently being tested alone and in combination with other agents in multiple phase I solid tumor and leukemia clinical trials, including a trial in adolescents and young adults [8]. To further evaluate the therapeutic potential of MERTK inhibition in EWS, we tested the activity of MRX-2843 against EWS cell lines. MRX-2843 mediated potent and dose-dependent decreases in MERTK activation, downstream oncogenic and apoptotic signaling pathways, cell density, and colony-forming potential in EWS cell lines. As combination therapies are more likely to be effective and provide sustained anti-tumor activity, we tested the combination of MRX-2843 with BCL-2 family inhibitors. We and others have demonstrated regulation of BCL-2 downstream of MERTK. NSCLC cells treated with MERTK inhibitors had decreased expression of anti-apoptotic BCL-2 and SURVIVIN proteins [22,35]. NSCLC cells with shRNA-mediated MERTK knockdown had reduced pro-survival AKT/CREB signaling and reduced levels of SURVIVIN and BCL-XL [14]. AKT, a component of the PI3K/AKT pathway activated by MERTK, is known to promote the expression of BCL-2 [36]. EWS-FLI1 also induces BCL-2 expression [23]. These findings suggest that combined MERTK and BCL-2 family inhibition may be particularly effective in EWS. Here, treatment with MRX-2843 and venetoclax (BCL-2 inhibition) or navitoclax (BCL-2/BCL-XL) provided enhanced therapeutic activity against EWS cell lines compared to single agents. Mathematical modeling revealed primarily additive and some synergetic interactions between MRX-2843 and BCL-2 family inhibitors. Similar studies to assess interactions between MRX-2843 and other classes of therapies that are known to be active against EWS should be completed to determine the best combination for clinical testing. These studies may identify combinations with more consistently synergistic interactions that are more easily translatable to clinical studies in patients with EWS.

Venetoclax is a BCL-2-selective small-molecule inhibitor that is FDA-approved in combination with other agents for the treatment of adults with chronic lymphocytic leukemia, small lymphocytic lymphoma, or AML [24]. Clinical studies are also investigating venetoclax in pediatric populations in combination with cytotoxic chemotherapy [37]. Navitoclax targets BCL-2 and BCL-XL and has been widely examined in combination with other therapies in numerous solid tumors, including rhabdomyosarcoma, small cell lung cancer, and endometrial carcinoma, and in hematologic malignancies including AML and ALL [38]. Treatment with either venetoclax or navitoclax in combination with cytotoxic chemotherapy has resulted in dose-limiting thrombocytopenia and/or neutropenia [37,39]. The combination of two targeted therapies to inhibit MERTK and BCL-2, however, may be better tolerated.

The interactions between MRX-2843 and BCL-2/BCL-XL inhibitors demonstrated here were potent and consistent across multiple agents, cell lines and drugs that are currently approved or have been tested extensively in clinical trials, including trials in children and young adults. Similarly, BCL-2 inhibition with venetoclax increased sensitivity to nab-paclitaxel in highly resistant BCL-2-expressing EWS xenografts [40]. Future studies will also explore strategies targeting BCL-XL and MCL-1 in combination with MERTK inhibition. The EWS-FLI1 fusion protein promotes expression of MCL-1 [41]. As a result, cell lines expressing high levels of EWS-FLI1 are relatively resistant to the BCL-2/BCL-XL inhibitor navitoclax. Derivative cell lines expressing doxycycline-inducible shRNA against the EWS-FLI1 fusion protein downregulate MCL-1 and are more sensitive to navitoclax. In contrast, EWS cells have similar sensitivity to obatoclax, a pan-BCL2 family inhibitor that targets BCL-2, BCL-XL and MCL-1, irrespective of how much EWS-FLI1 they express. These data indicate that EWS cells can be particularly dependent on MCL-1 for survival, especially if they express high levels of EWS-FLI1. Other studies have shown that pediatric solid tumors are more dependent on BCL-XL and MCL-1 than BCL-2, and that the dual inhibition of BCL-XL and MCL-1 provides synergistic anti-tumor activity in EWS cell line cultures [29]. Combined BCL-2 and BCL-XL inhibition, but not BCL-2 inhibition alone, also sensitized EWS cells to PARP inhibition [23]. Thus, while treatment with MRX-2843 combined with venetoclax or navitoclax provided robust anti-tumor activity in the studies reported here, the combined inhibition of BCL-XL, MCL1 and MERTK may provide even further improvement in therapeutic efficacy against EWS.

## 5. Conclusions

Our findings support the development of MRX-2843, alone and in combination with BCL-2 family inhibitors for treatment of EWS. MRX-2843 monotherapy is being tested in adults with relapsed/refractory, advanced, and/or metastatic solid tumors (NCT03510104), and an ongoing trial in relapsed/refractory acute leukemia (NCT04872478) has enrolled patients down to age 12, paving the way for a future trial in adolescents and adult patients with EWS.

## Data Availability

The data presented in this study are available for bona fide researchers on request from the corresponding author.

## Supplementary Materials

The following supporting information can be downloaded at: https://www.mdpi.com/article/10.3390/cancers16162831/s1, Figure S1: MERTK CERES dependency scores in EWS cell lines. Figure S2: EWS cell lines are not functionally dependent on TAM family receptors TYRO3 and AXL or TAM family ligands GAS6, PROS1, and LGALS3. CERES gene dependency scores were determined using CRISPR-CAS9 knockout data from the Broad Institute’s Cancer Dependency Map (DepMap) portal Avana 20Q4 database. Figure S3: Combined treatment with MRX-2843 and BCL-2 inhibitors provides enhanced therapeutic efficacy compared to single agents in Ewing sarcoma cell cultures.
