# Supplementary material for: MERTK Is a Potential Therapeutic Target in Ewing Sarcoma
Source: Cancers (Basel). 2024 Aug 12;16(16):2831. doi: 10.3390/cancers16162831 (PMC11352666; doi:10.3390/cancers16162831)
Supplement: Supplementary file 1 [file cancers-16-02831-s001.zip › cancers-2953578-supplementary.pdf]

Supplementary Figures

More essential

Cell Line	CERES
TC106	-0.707
A673	-0.556
TC32	-0.555
TC71	-0.450
SKES1	-0.380
CHLA57	-0.374
SKNMC	-0.319
TC138	-0.312
TC205	-0.311
COGE352	-0.252
SKNEP1	-0.248
RDES	-0.209
EWS502	-0.112
EW8	-0.034
SKPNDW	0.006

Figure S1. MERTK CERES dependency scores in EWS cell lines.

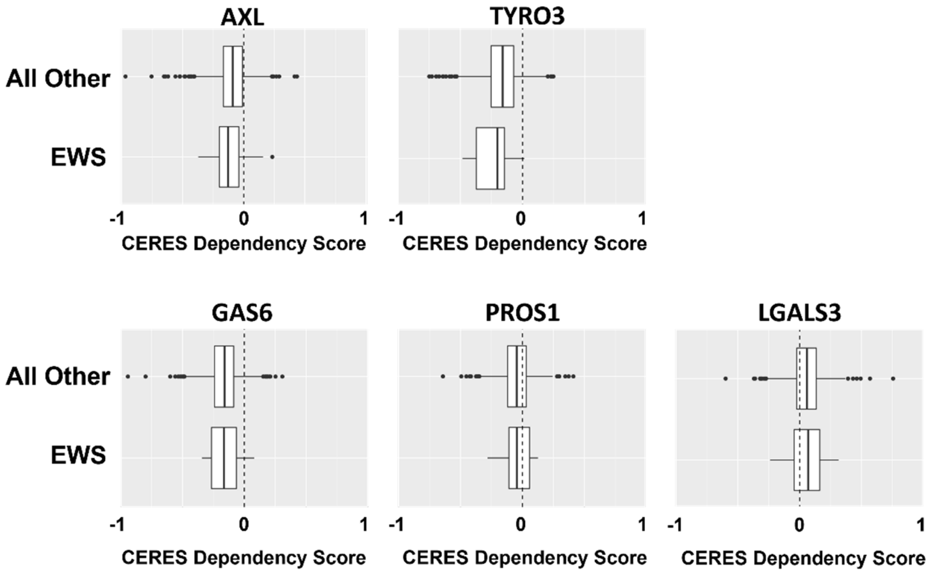

Figure S2. EWS cell lines are not functionally dependent on TAM family receptors TYRO3 and AXL or TAM family ligands GAS6, PROS1, and LGALS3. CERES gene dependency scores were determined using CRISPR-CAS9 knockout data from the Broad Institute's Cancer Dependency Map (DepMap) portal Avana 20Q4 database. A lower CERES score indicates greater functional dependence, with a score of -1 indicating an essential gene and a score of 0 indicating no significant reduction in cell density. Data are shown as box plots with median +/- quartile 1 and quartile 3. CERES dependency scores do not demonstrate enhanced dependence on TAM family members TYRO3 or AXL or TAM family ligands in EWS cell lines (n=16) relative to all other cell lines tested (n=773, p >0.05, Wilcoxon rank-sum test).

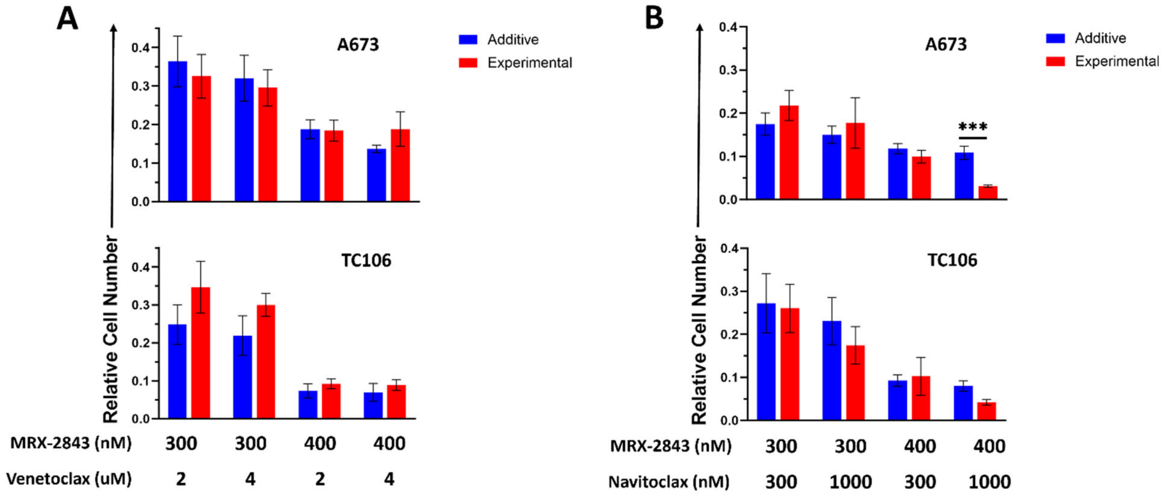

Figure S3. Combined treatment with MRX-2843 and BCL-2 inhibitors provides enhanced therapeutic efficacy compared to single agents in Ewing sarcoma cell cultures. (A) A673 or (B) TC106 cells were treated with the indicated concentrations of MRX-2843, venetoclax (VEN), navitoclax (NAV), MRX-2843 combined with VEN or NAV, or vehicle and relative cell densities were determined as in Figure 6. The fractional product method was used to calculate expected cell densities in cultures treated with combination therapies assuming an additive interaction between agents (Additive). Synergistic and antagonistic interactions are defined by significant decreases or increases, respectively, in cell density in cultures treated with the combination compared to the expected additive value. Mean +/- SEM were derived from at least three independent experiments (***p<0.001, unpaired t test).

Supplementary Materials – Immunoblots

Corresponding whole blots are depicted after cropped replicates.

Figure 2C – cropped replicates

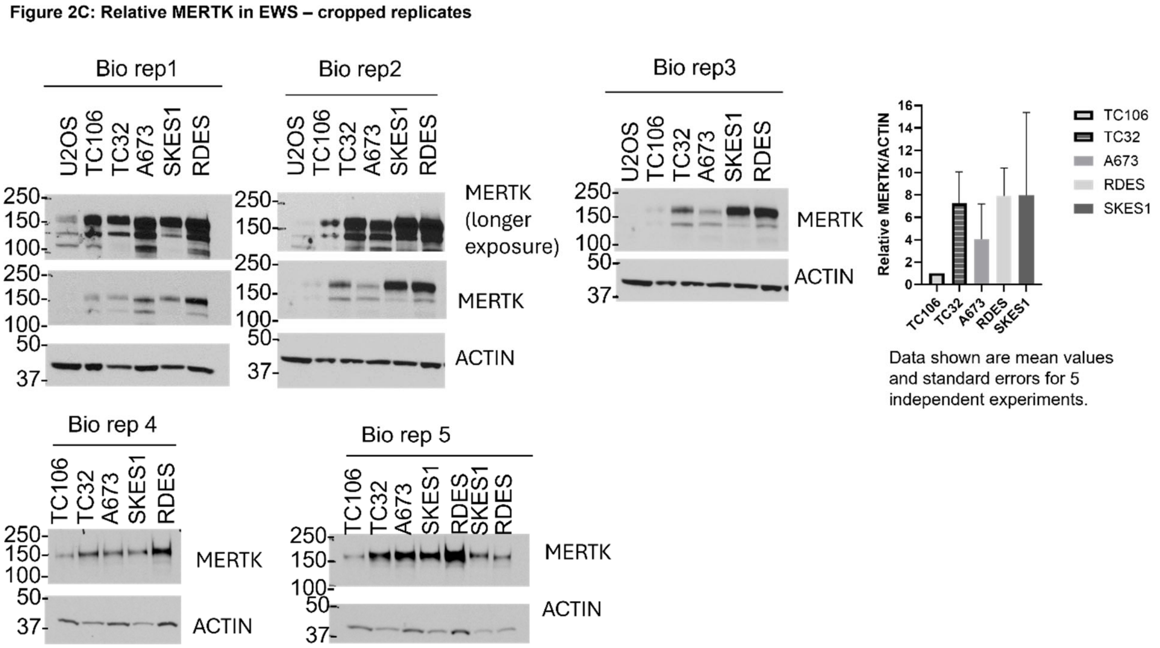

Figure 2C: Relative AXL in EWS – cropped replicates

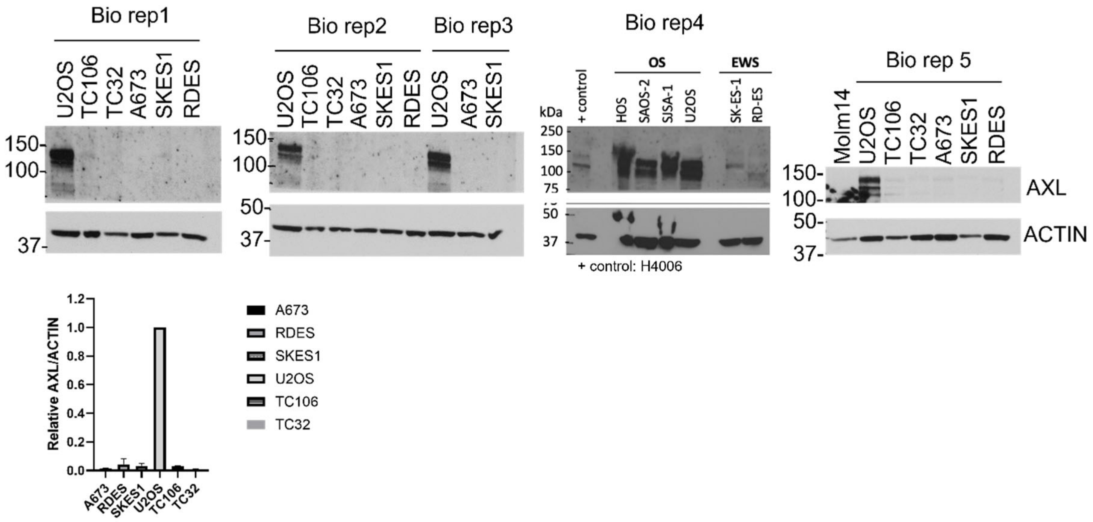

Data shown are mean values and standard errors from 3 - 5 independent experiments.

Figure 2C: Relative TYRO3 in EWS – cropped replicates

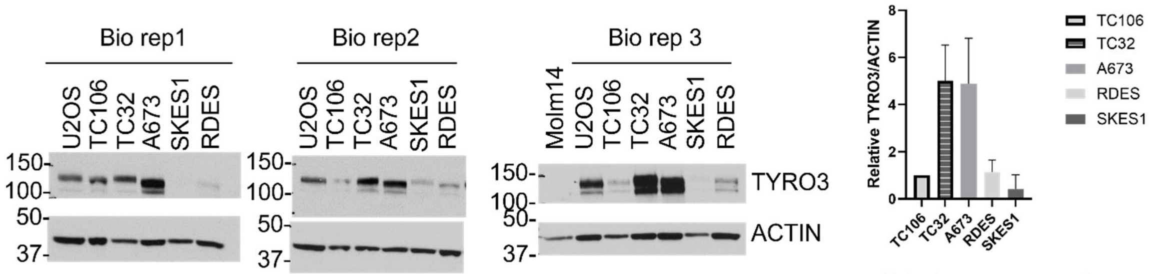

Data shown are mean values and standard errors for 3 independent experiments.

Figure 2C: Relative PROS1 in EWS – cropped replicates

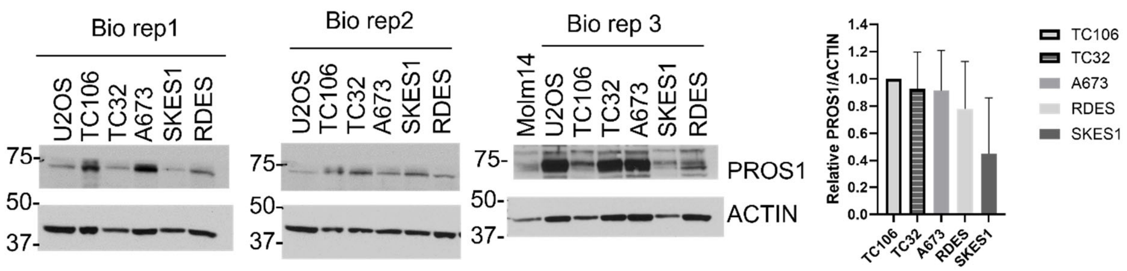

Data shown are mean values and standard errors for 3 independent experiments.

Figure 2C: Relative LGALS3 in EWS – cropped replicates

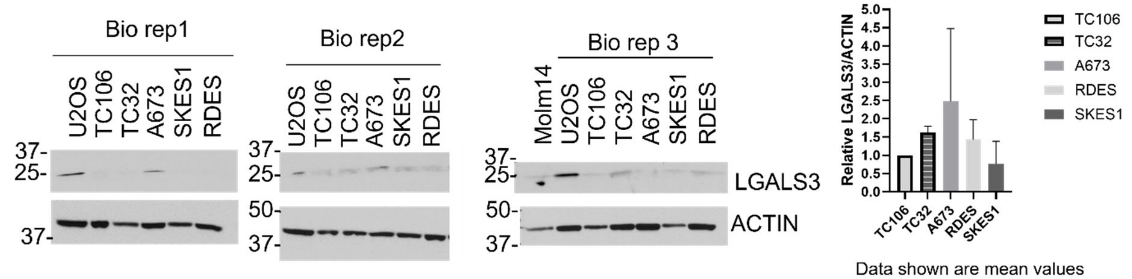

Data shown are mean values and standard errors for 3 independent experiments.

Figure 2C – whole blots

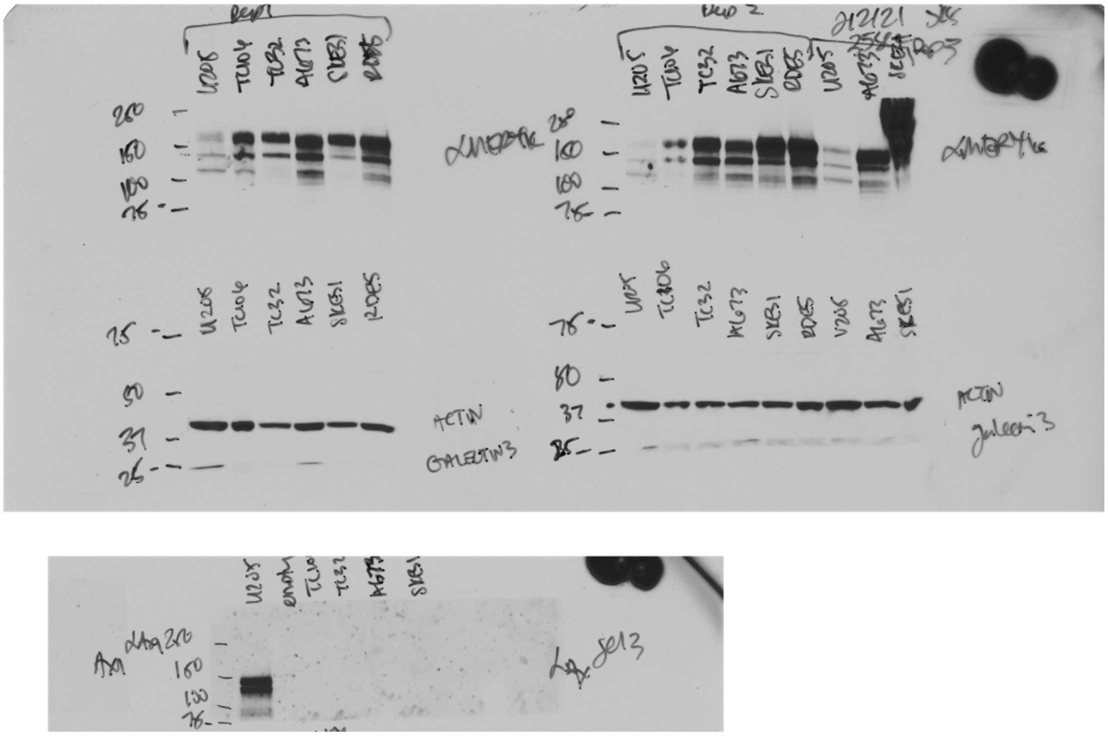

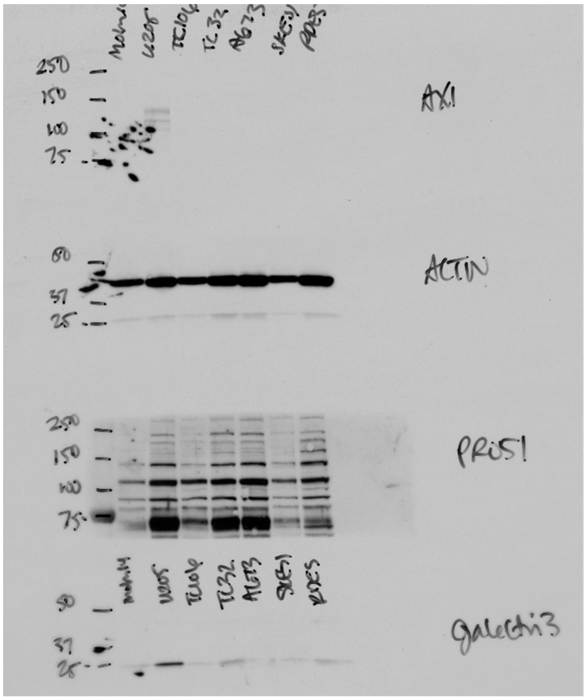
AXl
ACTIN
Galectin3

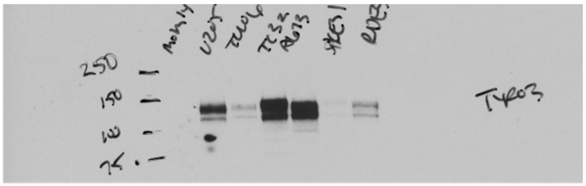
TYRO3

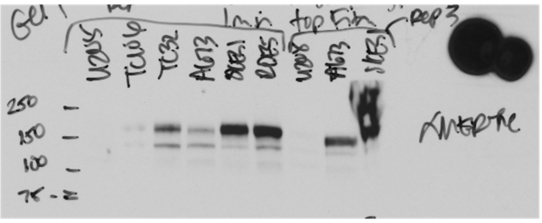
MERTK

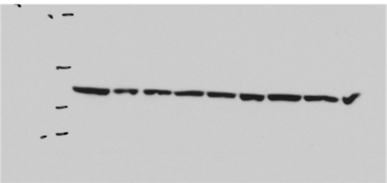

ACTIN

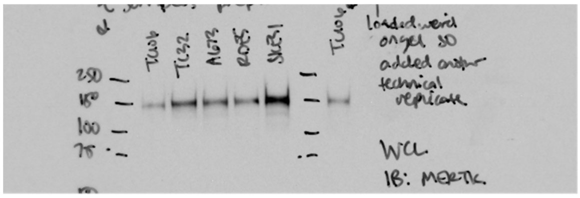
WCL
IB: MERTK

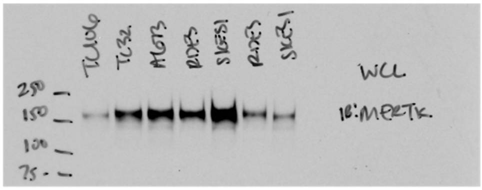
WCL
IB: MERTK

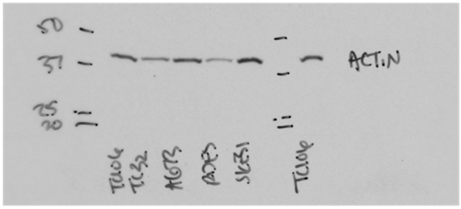
ACTIN

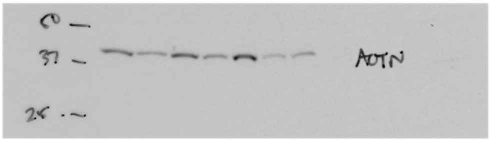
ACTIN

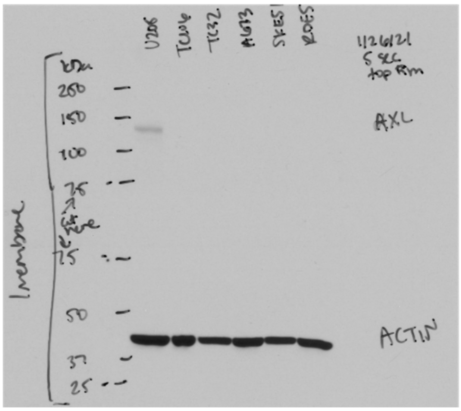
AXL
ACTIN

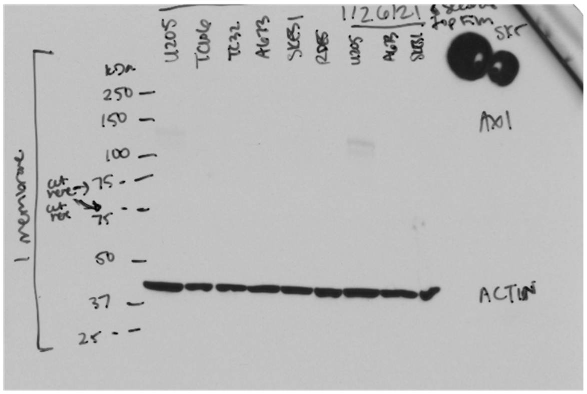
AXl
ACTIN

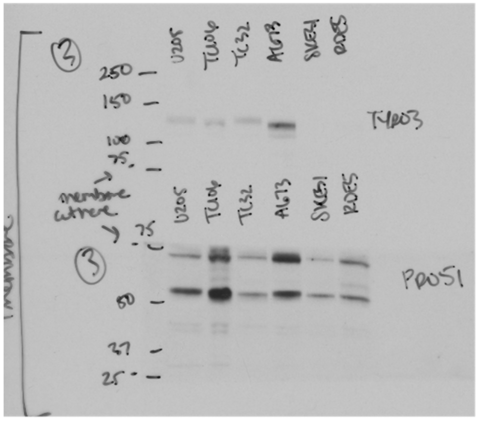

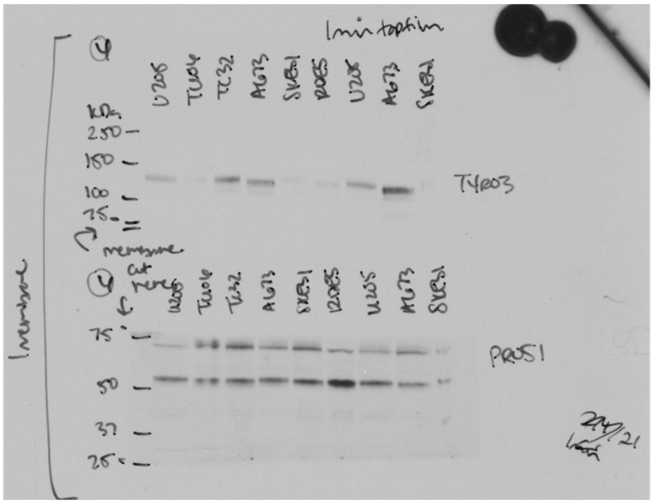

Figure 4A

Figure 4A: Relative pMERTK / MERTK in EWS cell lines – replicate 1

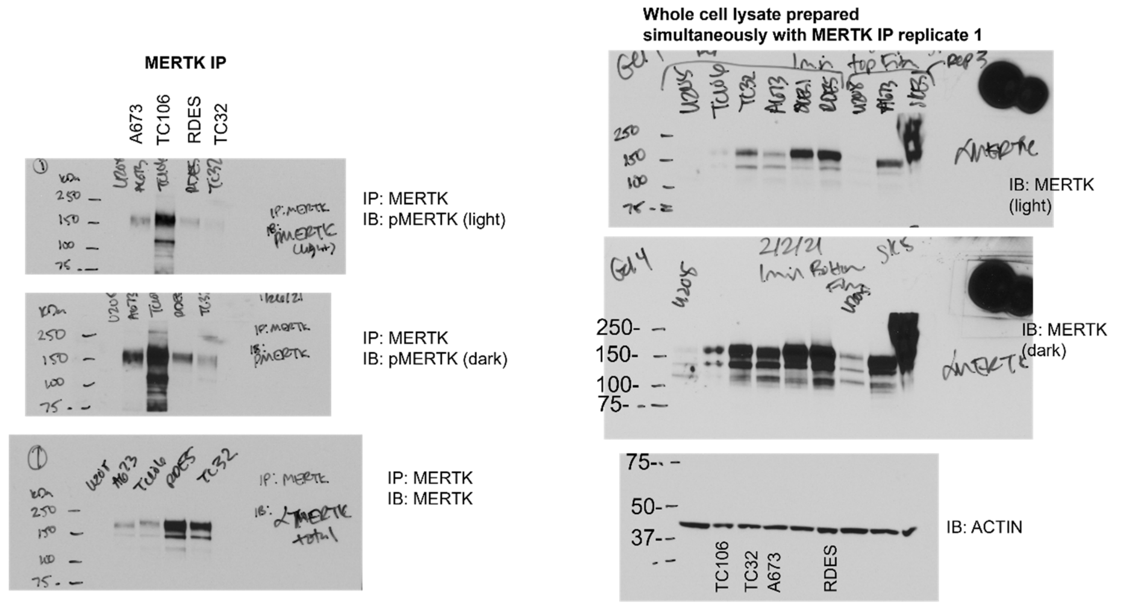

Figure 4A: Relative pMERTK/MERTK – replicate 2

MERTK IP

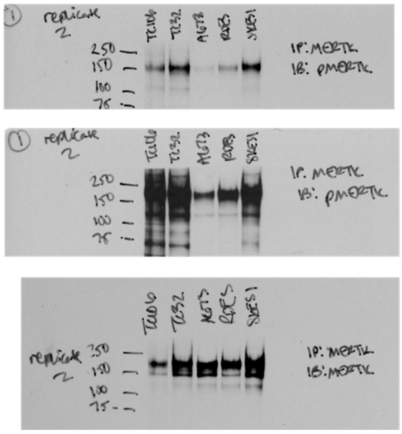

IP: MERTK
IB: pMERTK (light)

IP: MERTK
IB: pMERTK (dark)

IP: MERTK
IB: MERTK

Whole cell lysate prepared simultaneously with MERTK IP replicate 2

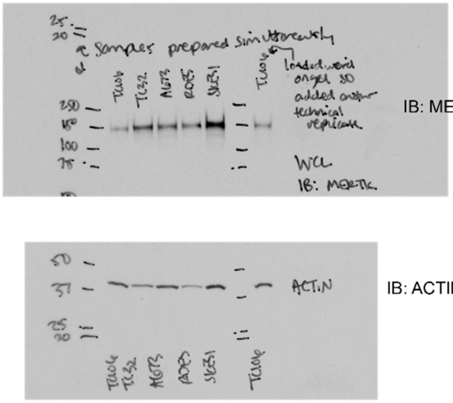

IB: MERTK

IB: ACTIN

Figure 4A: Relative pMERTK/MERTK – replicate 3

MERTK IP

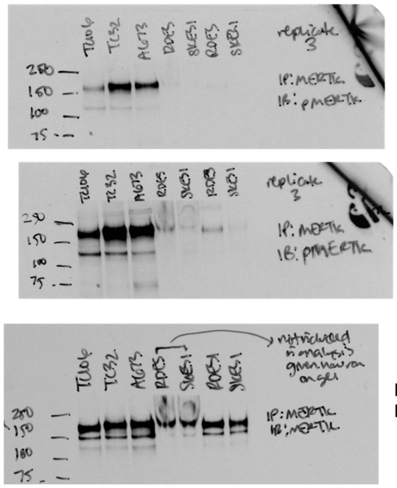

IP: MERTK
IB: pMERTK (light)

IP: MERTK
IB: pMERTK (dark)

IP: MERTK
IB: MERTK

Whole cell lysate prepared simultaneously with MERTK IP replicate 3

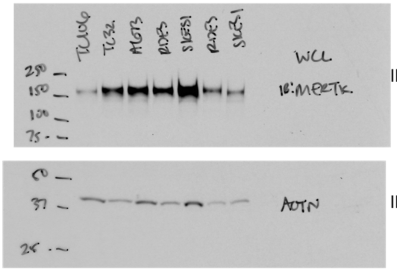

IB: MERTK

IB: ACTIN

Figure 4A: Densitometry analysis of blots

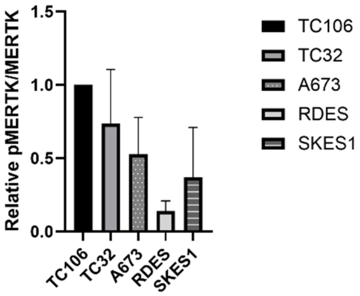

Data shown are mean values and standard errors for two (SKES1) or three (all others) independent experiments.

Figure 4B

Figure 4B: A673 pMERTK/MERTK with MRX-2843 treatment – cropped replicates

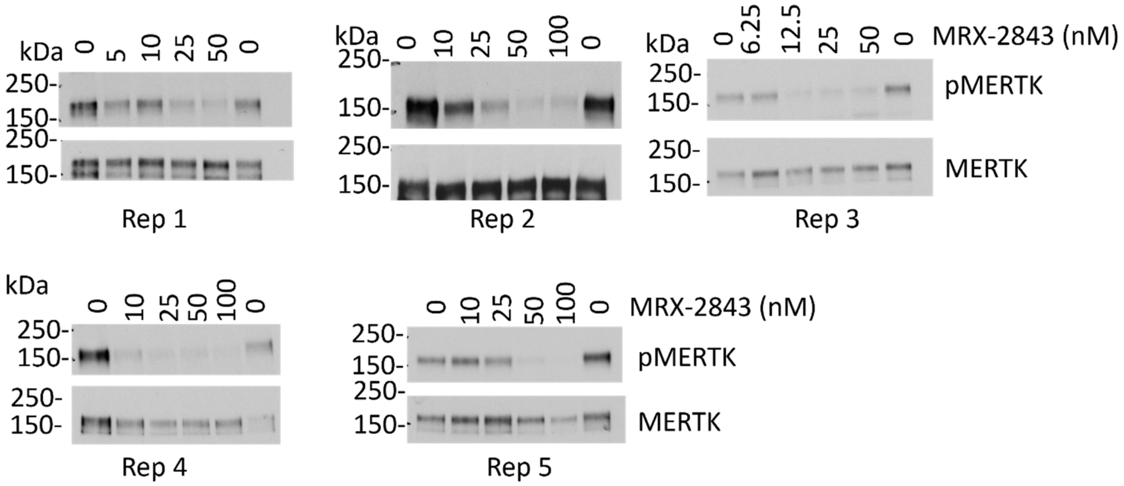

Densitometry analysis of blots is located in Figure 4C

Figure 4B: A673 pMERTK/MERTK with MRX-2843 treatment – whole blots

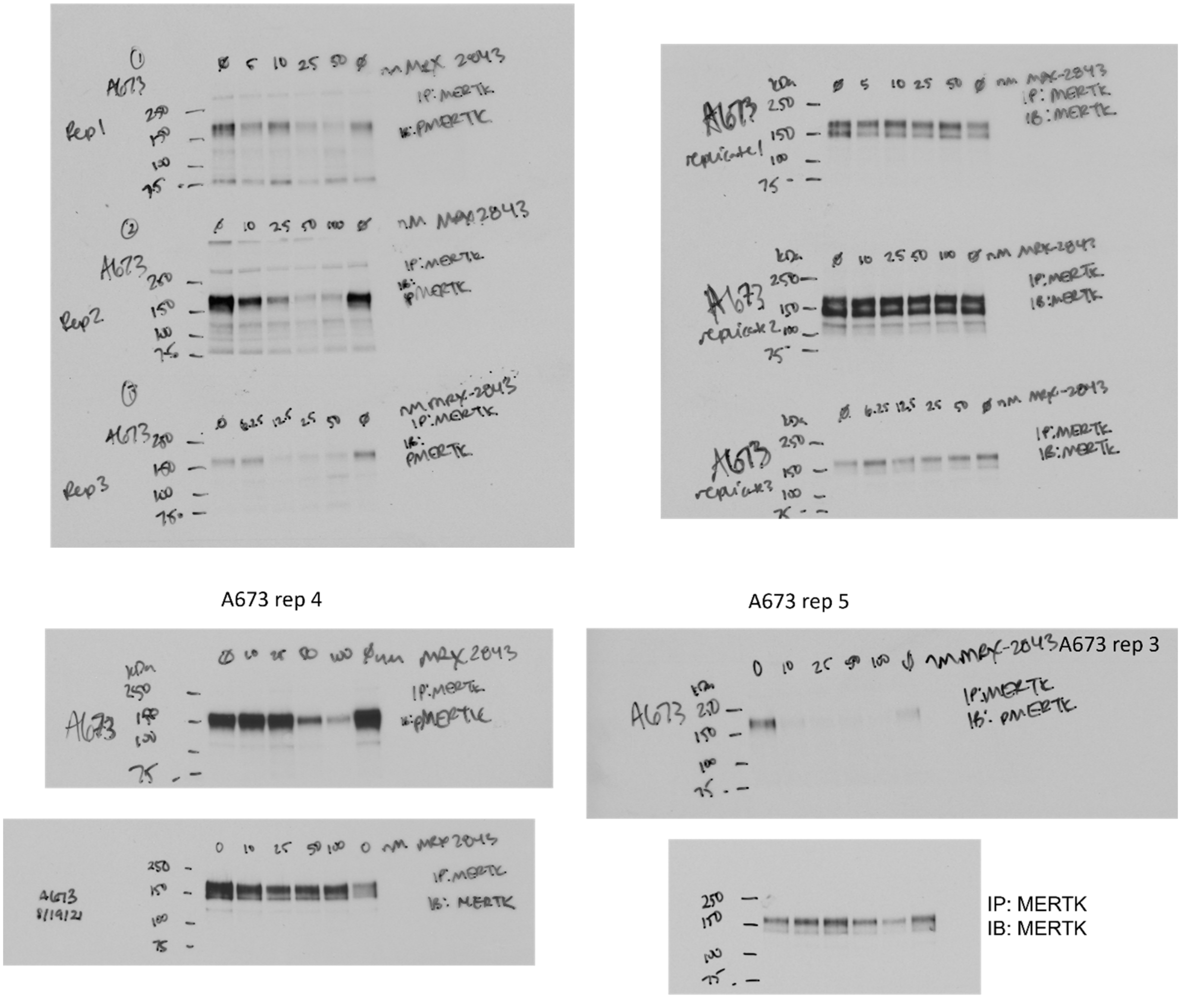

Figure 4B: TC106 pMERTK/MERTK with MRX-2843 treatment – cropped replicates

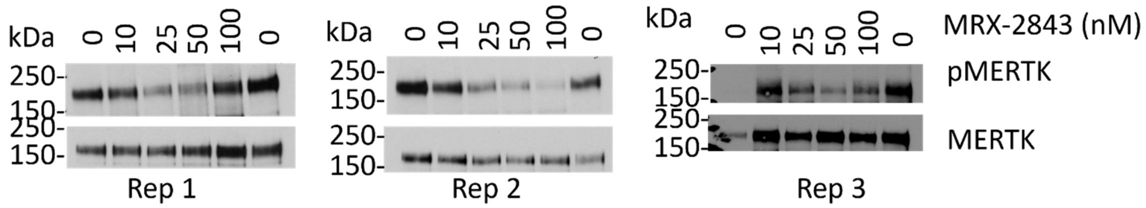

Densitometry analysis of blots is located in Figure 4C

Figure 4B: TC106 pMERTK/MERTK with MRX-2843 treatment

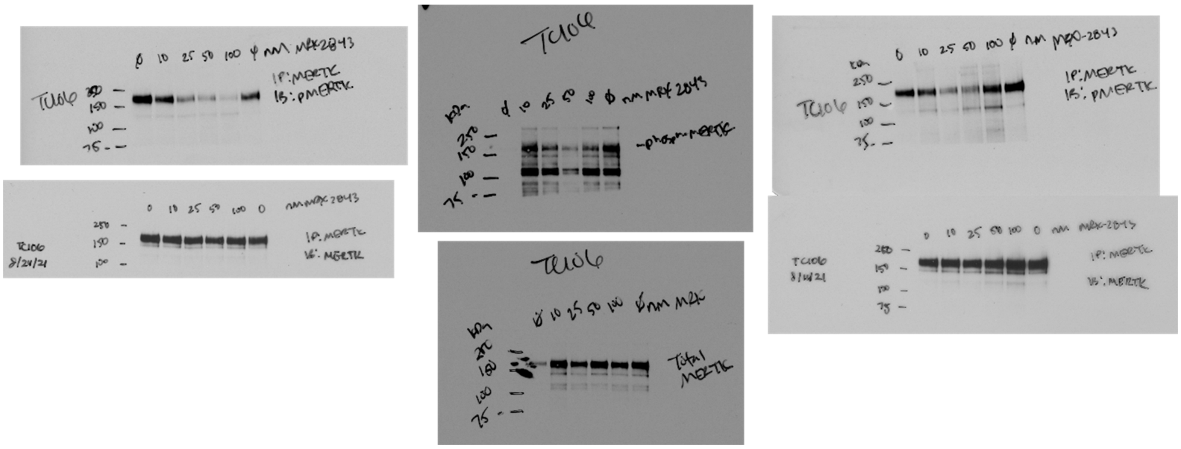

Figure 4D

Figure 4D: A673 cropped replicates – pERK /ERK and pSTAT6 / STAT6

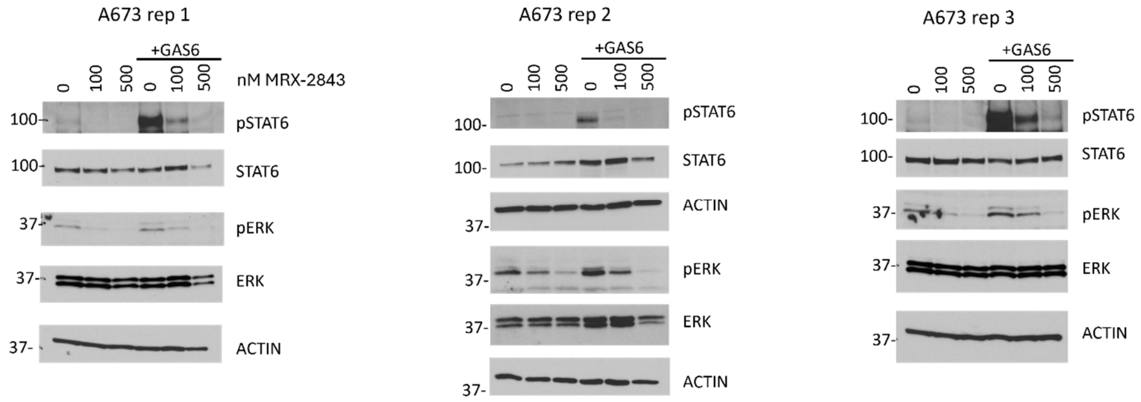

Densitometry analysis of blots is located in Figure 4E

Figure 4D: A673 pERK and total ERK – full blots

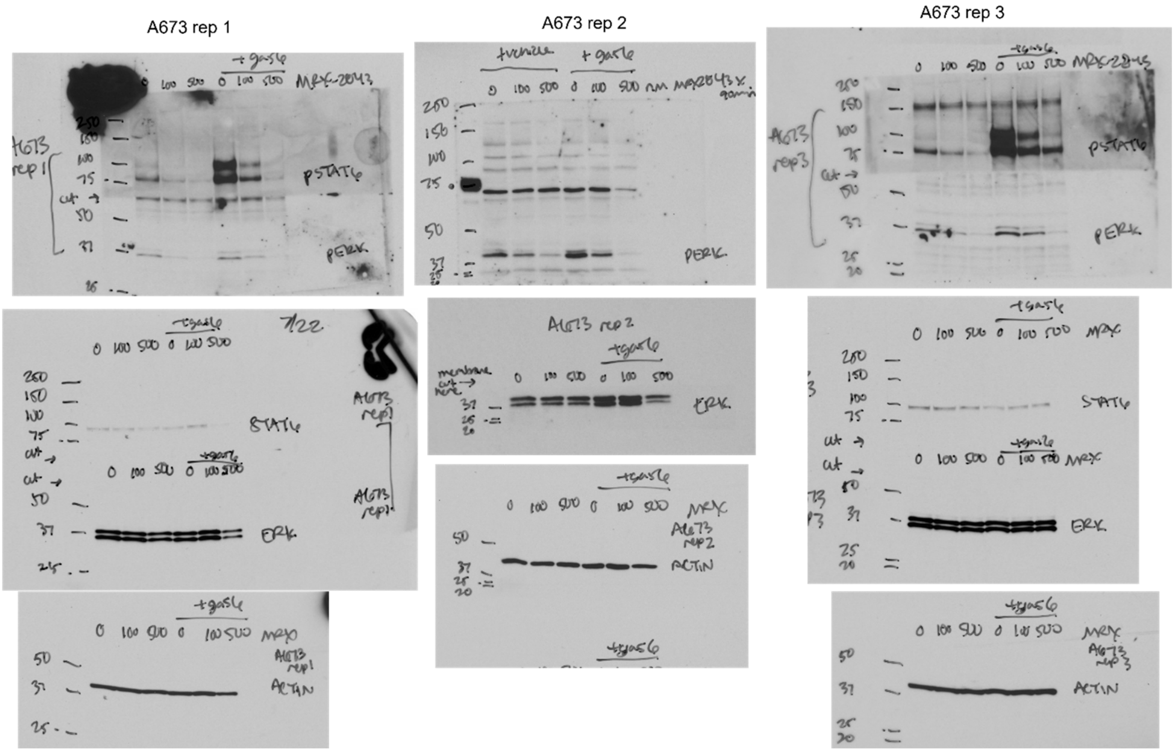

Figure 4D: A673 pSTAT6 and STAT6 – full blots

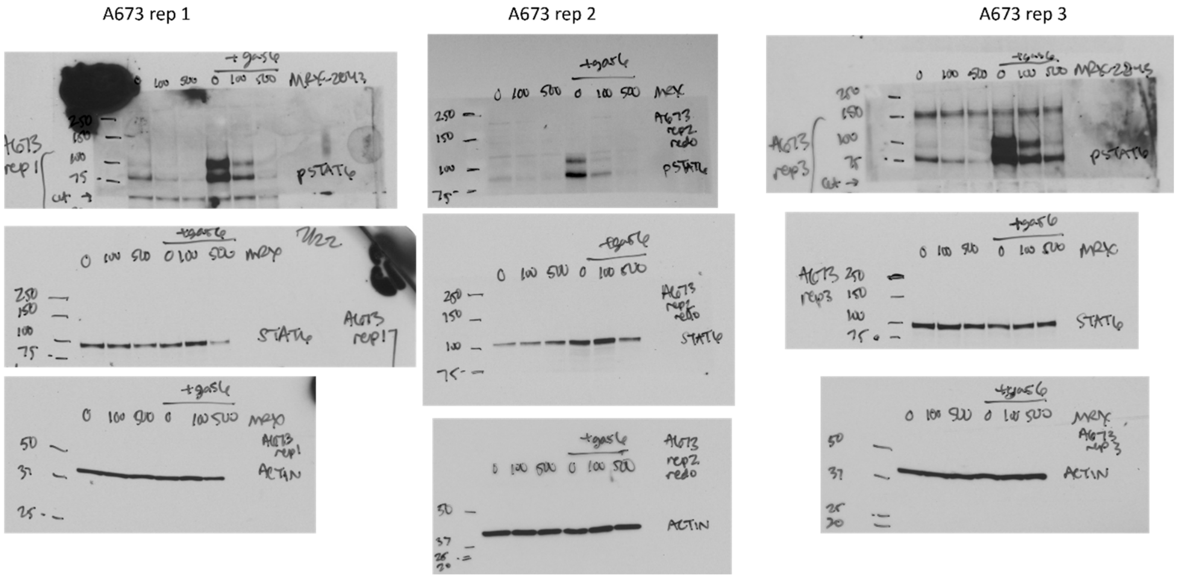

Figure 4D: TC106 cropped replicates – pERK and pSTAT6

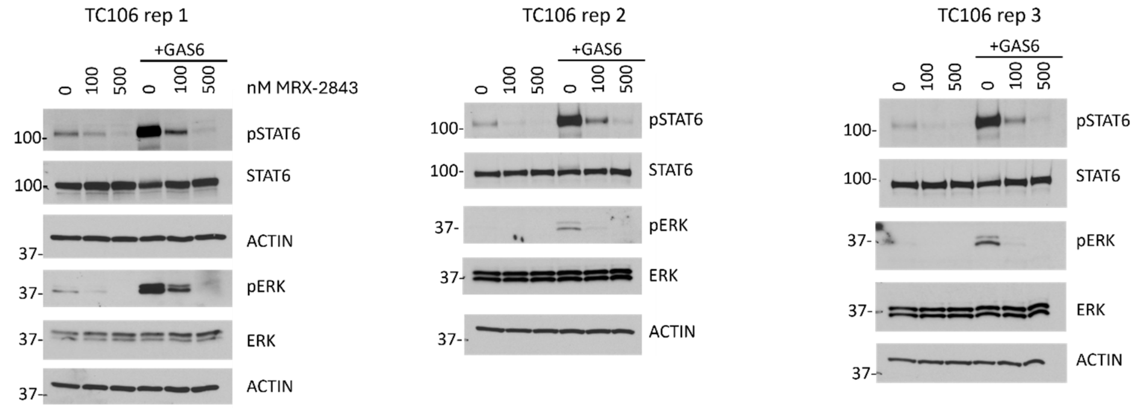

Densitometry analysis of blots is located in Figure 4E

Figure 4D: TC106 pERK and total ERK – full blots

TC106 rep 1

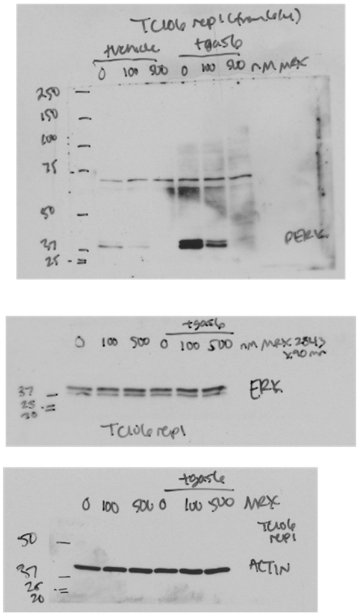

TC106 rep 2

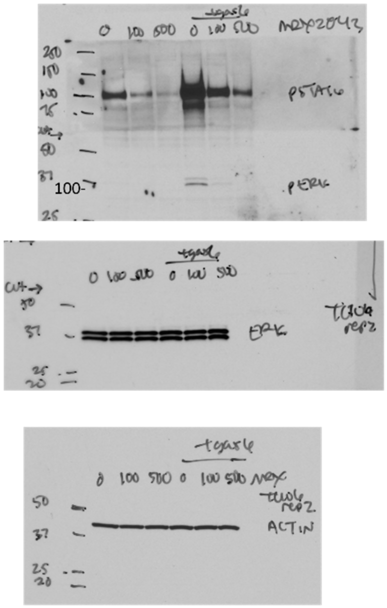

TC106 rep 3

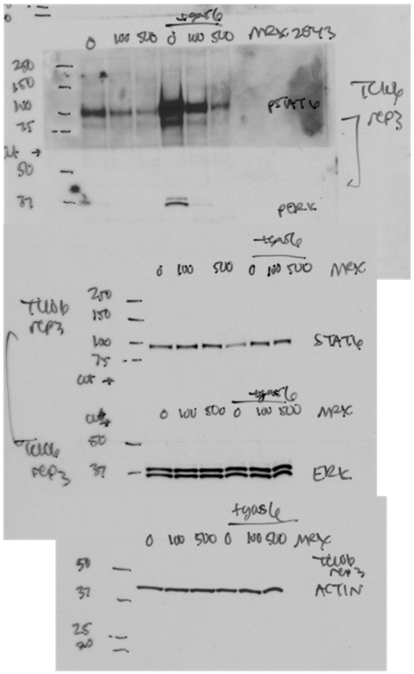

Figure 4D: TC106 pSTAT6 and STAT6 – full blots

TC106 rep 1

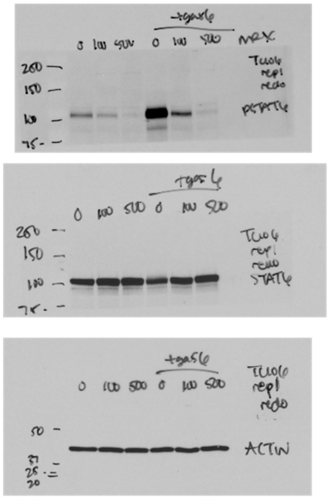

TC106 rep 2

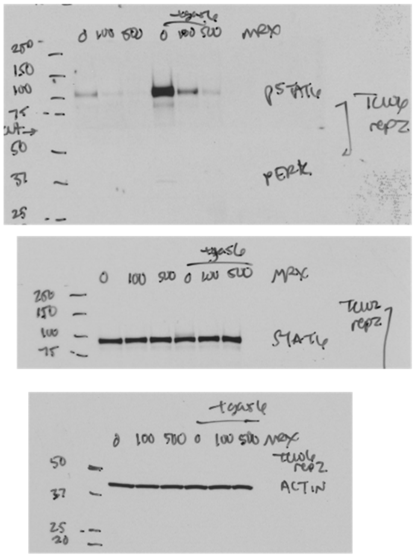

TC106 rep 3

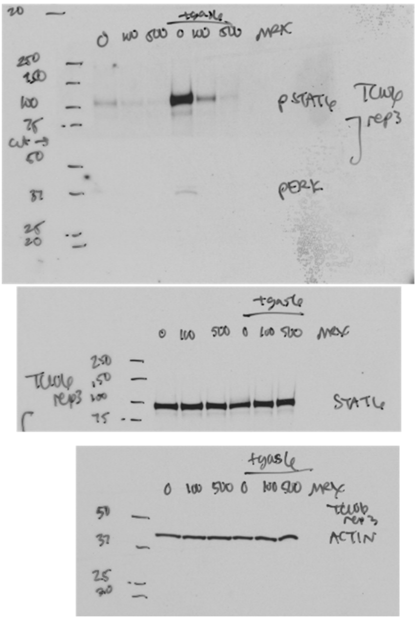

Figure 4F

Figure 4F: A673 SURVIVIN – cropped replicates and full blots

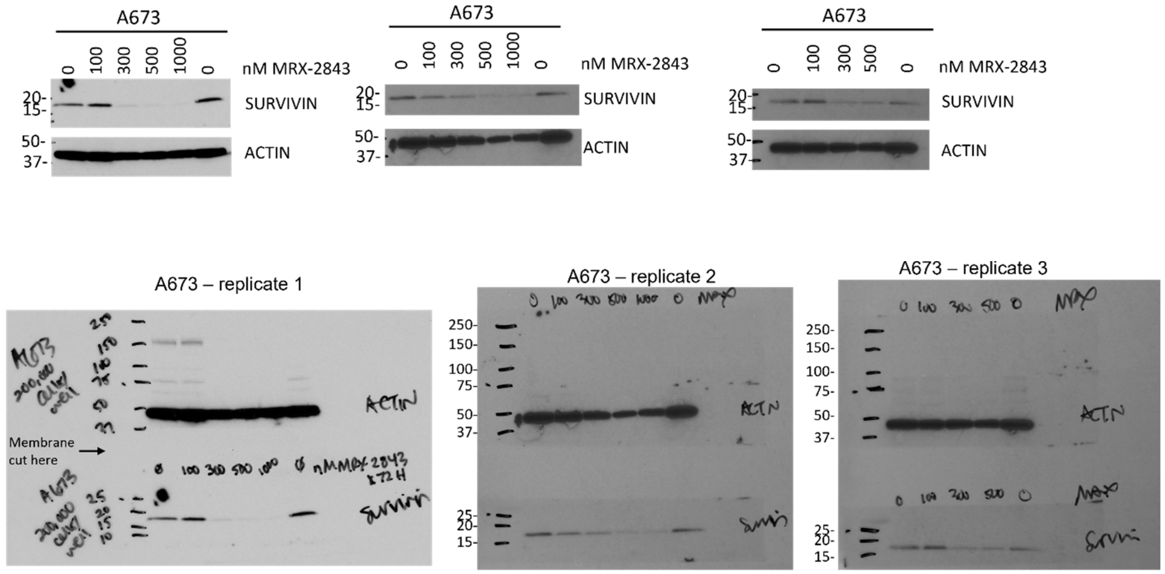

Figure 4F: TC106 SURVIVIN – cropped replicates and full blots

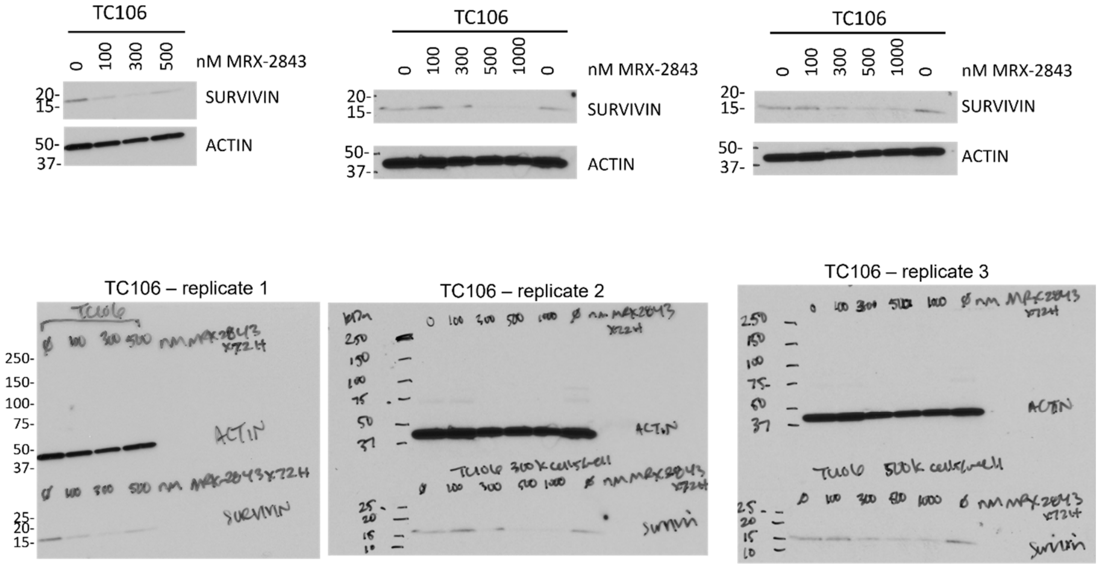

Densitometry analysis of blots is located in Figure 4G
